# A dataset of EEG recordings from 47 participants collected during a virtual reality working memory task where attention was cued by a social avatar and non-social stick cue

**DOI:** 10.1016/j.dib.2022.107827

**Published:** 2022-01-15

**Authors:** Samantha E.A. Gregory, Hongfang Wang, Klaus Kessler

**Affiliations:** a Department of Psychology, University of Salford, UK; b Institute of Health and Neurodevelopment, Aston Laboratory for Immersive Virtual Environments, Aston University, UK; c School of Psychology, University College Dublin, Republic of Ireland

**Keywords:** Eye gaze, Social, Joint attention, Theta, Alpha, Neural oscilations

## Abstract

This data article describes electroencephalography (EEG) and behavioral data from 47 participants. Data was collected using a 64 channel eego™ sports mobile EEG system during a visual working memory task presented in virtual reality (VR) using Unity with an Oculus Rift S head-mounted display. In the memory task, participants had to remember the status of and details about objects presented on a table. Prior to object appearance a moving, 3D social avatar or non-social stick cue was presented which pointed to the left or right of the table. Items for encoding could appear in the valid, cued location or the invalid, un-cued location, with the cue being uninformative to the task. The cue type was presented within subjects, blocked, counterbalanced. The data (behavioral & EEG (raw and processed)), scripts and the full task are available. The data is set up to allow investigation of neural signals during attention cueing, and memory encoding, maintenance and retrieval. The main novelty of the dataset is the presentation of the social avatar and non-social stick cue in VR within subjects, thus, allowing comparison across time points, including a period of eye contact. The data is also of interest to researchers interested in the neural corelates of working memory. Further, it is of interest to researchers interested in combining VR and EEG.

## Specifications Table

**Table:** 

Subject	Neuroscience: Cognitive
Specific subject area	Neuro VR (EEG), Working memory, social cuing
Type of data	Raw and processed EEG data Analysis scripts (Matlab® code) Behavioral data (CSV)
How the data were acquired	EEG data were acquired using a 64 channel eego™ sports mobile EEG system (ANT Neuro, Enschede, The Netherlands; Ag/AgCl electrodes, international 10–10 system), digitised at a sampling rate of 500Hz. Mastoids and EOG electrodes were not used, electrode CPz served as online reference and AFz as the ground electrode. Impedance was kept below 20 kΩ during task. The EEG marker signals (to denote the trial events) were sent from the testing PC to the tablet recording the EEG data using the lab stream layer (https://github.com/labstreaminglayer/LSL4Unity). Behavioural data were acquired through Unity (unity.com/) using the Unity experimental framework [1] run on a Lenovo Legion Y540-17IRH laptop computer (Intel Core i7-9750H Processor, 32 GB RAM, NVIDIA GeForce RTX 2060 graphics card) which presented the study via the Oculus Rift S PC-Powered VR Gaming Head-Mounted Display (HMD) and collected responses from a touch controller. The study and materials can be downloaded from the OSF: https://osf.io/s9xmu/files/.
Data format	Raw: BIDS format (.eeg, .vhdr, .vmrk) Pre-processed: Matlab® .mat Behavioural: .csv
Description of data collection	There were 10 practice and 112 experimental trials per cue type (social and non-social, blocked, counterbalanced) presented in VR using the HMD. The cue was presented at centre with memory items presented laterally. The task was to remember the objects and ignore the cue. Breaks were encouraged every 28 trials and enforced between cue types. The HMD could be removed during breaks.
Data source location	• Institution: Aston University • City/Town/Region: Birmingham • Country: UK • Latitude and longitude (and GPS coordinates, if possible) for collected samples/data: 52.48734822158862, -1.8898354346688258
Data accessibility	Repository name: OpenNeuro Data identification number: doi:10.18112/openneuro.ds003702.v1.0.1 Direct URL to data: openneuro.org/datasets/ds003702 Additional data including avatar ratings and data from an online version of the study, the pre-registration and the full task is available on the OSF: https://osf.io/s9xmu/files. There are no access controls.
Related research article	*S. E. A, Gregory, H. Wang, K. Kessler, EEG alpha and theta signatures of socially and non-socially cued working memory in virtual reality, Social cognitive and affective neuroscience. (2021)* nsab123, https://doi.org/10.1093/scan/nsab123

## Value of the Data

• This dataset allows the direct comparison of dynamic social avatar and non-social cues in VR with EEG, including the influence of eye contact and movement.
• The data is of interest to researchers investigating the neural corelates of working memory, the experiment required participants to remember details both about the location and status of the objects, and marker codes allow assessment of the EEG data during encoding, maintenance and retrieval, with separate retrieval of the status and location information.
• The data is of interest to researchers who are conducting research combining VR and EEG. The VR headset used is a widely available commercial headset and the EEG system uses patented shielding technology with passive electrodes allowing use of the system in noisy environments without a faraday cage. Therefore, the data can be used to assess this system.
• The social avatars used can be assessed independently of the memory task. Prior to the memory display the avatars were presented looking down before looking up to make eye contact with the participants. They hold eye contact for 1000ms. The avatars used have been independently rated (not by the participants in the study) for key traits, with this data available on the OSF: https://osf.io/h89tz/. Four male and four female avatars were used, allowing for comparison of response to avatar gender. Further, avatars presented showed neutral facial expressions, and so brain dynamics in response to these neutral stimuli may be used as a benchmark against which researchers can compare responses to non-neutral avatar stimuli – the avatars themselves are included in the experiment package on the OSF: https://osf.io/fw8n6/.
• The provision of raw data allows researchers to process the data with different feature extraction and selection techniques, and the data has markers for key points in the study that have not been investigated in depth thus allowing for additional analyses than those presented in the related article [2].

## 1 Data Description

The data includes both behavioural and EEG data for 47 participants with full EEG and 49 behavioural only. Behavioural data consist of accuracy and reaction times for response to the location and status probes. The EEG data presented consists of both raw unprocessed data and data that has been processed. This data is hosted on the OpenNeuro platform (openneuro.org/datasets/ds003702) with the raw EEG data presented in BIDs format [3]. Matlab® analysis files (using the Fieldtrip toolbox version 20191028: https://www.fieldtriptoolbox.org/) are also presented on OpenNeuro in the code folder – these are saved as txt files for those without Matlab® access to easily view the code, for Matlab®, the file extension needs to be changed to .m. Supplementary material on the OSF (https://osf.io/s9xmu/files) consists of data from the online version of the study (experiment 2 in Gregory et al., 2021), trait ratings for the avatars (from an online 2D ratings study), and the full experimental protocol (a zipped unity file) which includes the avatars and the task.

### 1.1 Raw data

Data is presented in BIDS format (.eeg, .vhdr, .vmrk). This means that for each participant there is an individual file which contains a tsv file which has the channels recorded (same for each participant), and then the data in brain vision format (https://pressrelease.brainproducts.com/bids/) which consists of: 1. The binary data file (.eeg) containing the recorded time series of the EEG; 2. The header file (.vhdr), containing information about the data including the amplifier settings, software filters, number of channels, impedance values and references to the .eeg and .vmrk files; 3.The marker file (.vmrk) which lists the markers present in the EEG data as well as their data point position, timestamps, marker type, description, and reference to the .eeg file (markers are with details are also listed on OpenNeuro in the code file:  ReadMe-EventCodes.txt). This data can be processed using the Matlab® analysis scripts included in the code folder on OpenNeuro or using your own code in platforms such as Matlab® and EEGLAB [4].

### 1.2 Processed

The processed data is in Matlab® .mat format. This is presented in OpenNeuro in the derivatives folder and then in the EEGPreprocessedDataTableStudy folder. Each participant's data is presented as a zip file. In each file, the filename is always data_ica relating to the fact that this is the data post the independent components analysis conducted for data cleaning (see EEG pre-processing below). This data needs to be downloaded and saved in the separate folders for each participant (as presented) and can be loaded and analysed using the analysis script (Analysis_MatlabScript_Fieldtrip) in the code file.

### 1.3 Behavioural

The behavioural data from the VR experiment is presented on OpenNeuro in the *BehavioralData_TableStudyVR* folder. This contains the processed data in .csv format with a data dictionary explaining all file headers. In an additional folder: *TableStudyRawdata*, the raw data is found again with a data dictionary explaining the headings found in the csv files for each participant's raw data. For the raw data there is a separate file for each participant which contains 3 files: the data for the avatar and stick conditions separately (.csv format) and a participant details .json which contains age, gender and handedness information. Note that there are 49 behavioural data files, as there are 2 participants for which there is no EEG data – 1 due to recording failure, the other due to EEG not being conducted.

### 1.4 OSF supplements

The OSF repository contains: 1. The preregistration for the study presented in Gregory et al., (2021). 2. The experiment files for the VR version of the study, including the stimuli presented – these are presented in the *Experiment 1: Unity project virtual cuing social and non-social* folder. The Avatar and Stick projects are presented separately and are unity packages downloadable as zip files, these contain everything needed to run the project in Unity. 3. Behavioural data (raw and processed with data dictionaries) and the experiment file (a PsychoPy study) for Experiment 2, an online version of the study presented in Gregory et al (2021). 4. Videos of each of the cues presented as mp4s (in stimuli examples). 5. Link to a project where the virtual humans were rated online: Rating virtual humans. In the project, referenced in Gregory et al (2021), participants saw online videos of the avatars used in the study and rated them on human traits using 2 scales, the Godspeed which looks at human traits in avatars and robots [5] and a face trait questionnaire which was developed to investigate how people evaluate faces [6]. The study, developed in PyschoPy, and the data for both the trait and Godspeed questionnaire are presented – data is provided for each participant for each avatar in a .csv file with the rating for each trait presented. A read me file is also provided which explains the nature of the questionnaire. This supplementary data is not detailed further as part of this Data in Brief paper.

## 2 Experimental Design, Materials and Methods

### 2.1 Participants

For the EEG-VR study we recruited 49 participants (33 females, 16 males, mean age 21 years (SD = 3.1, range 18 – 32), 3 left-handed), with 47 of those providing EEG data (2 female participants did not have EEG data recorded). Participants received payment (£10/ hour, cash) or course credit and reported having normal or corrected to normal vision.

### 2.2 Apparatus

The experiment was run on a Lenovo Legion Y540-17IRH laptop computer (Intel Core i7-9750H Processor, 32 GB RAM, NVIDIA GeForce RTX 2060 graphics card), and presented through the Oculus Rift S PC-Powered VR Gaming Head-Mounted Display (HMD), with participants responding to stimuli using wireless touch controllers. The experiment was programmed in Unity using the Unity experimental framework [1] with triggers communicated wirelessly to the EEG (LSL4Unity; https://github.com/labstreaminglayer/LSL4Unity).

EEG was recorded using a 64 channel eego™ sports mobile EEG system (ANT Neuro, Enschede, The Netherlands; Ag/AgCl electrodes, international 10–10 system), digitised at a sampling rate of 500 Hz. For recording, the reference electrode was CPz and the ground was AFz. Impedance was kept below 20 kΩ during task and we did not set up to record data for the mastoids or EOG electrodes.

### 2.3 Stimuli

**Fig. 1:**
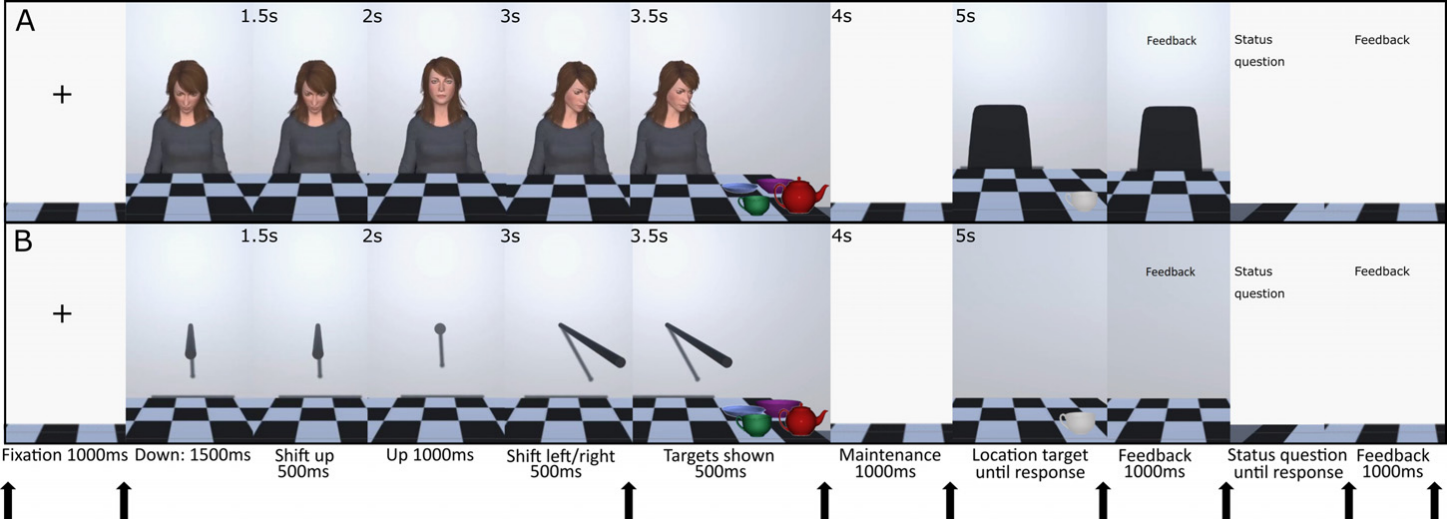
Illustration of the trial procedure showing the social avatar cue (panel A) and the non-social stick cue (panel B). Arrows denote the moments at which an EEG trigger was sent. Modified from Gregory et al [2].

**Human avatar cue:** Adobe Fuse (discontinued software) was used to create four male and four female identities showing neutral facial expressions and wearing plain grey clothing. Bone structure was added using Adobe Mixamo (www.mixamo.com), where the avatars were also placed in a seated position. The inbuilt animator in Unity was used to add head, neck and eye movement animations with no other body movements used. The Avatars were independently rated (n = 61, online study; https://osf.io/h89tz/) for human personality traits [6], as well as using the Godspeed questionnaire to assess anthropomorphism, animacy, and likeability [5]. Ratings from these questionnaires indicated that the avatars were humanlike. The avatars were presented as life sized in 3D via the HMD, such that the participant was sat across a table from them (see Fig. 1A).

**Non-social stick cue:** A cylindrical game-object which extended to a similar distance from the participant and table as the avatars was created in Unity. This was animated using the inbuilt Unity animator following the same trajectory as the human avatar cue (See Fig. 1B).

**Memory targets:** Target items were always a cup, bowl, teapot and pastry on a plate (i.e. items commonly found on a table). These were adapted from the Unity asset store (assetstore.unity.com/packages/3d/white-porcelain-dish-set-demo-82858; assetstore.unity.com/packages/3d/props/food/croissants-pack-112263), the cup could be empty or full of coffee, the bowl could be empty or full of soup, the plate could contain a pastry that was bitten or whole and the teapot was presented cracked or not cracked, these edits were made within Unity. To avoid colour matching at retrieval, the items were presented in colour at encoding and grayscale at retrieval. These were presented to be approximately life sized on the table in 3D (See Fig. 1).

### 2.4 Procedure

The participant's task was to remember the location and status details (i.e. whether the cup was full or empty) about targets presented on the table. The participants were informed that the cue (stick/ human avatar) was there to distract them and would not be helpful in the task. The table was topped with a checked pattern such that each item appeared in a single square at encoding. The location probe item was then presented in grayscale in either the same square at retrieval, or in a square which another item had occupied at encoding. The status probe consisted of a question, presented on a white background (e.g. ‘Did the bowl have soup in it?’). Participants were given feedback on every trial for both the location and status questions separately, with this presented for 1000 ms.

To configure the HMD and allow the participant to become familiar with the virtual environment and the response buttons on the touch controller there was a 5-trial familiarization session prior to the EEG set up. Once EEG was set up participants were given instruction on how to adjust and remove the HMD without moving the EEG cap, and the experimenter was present throughout to help with this. The study was free viewing and so participants could move their heads and eyes but were instructed to try to relax and avoid too much movement to ensure quality of the EEG.

For both cue types there were 10 practice trials and 112 experimental trials, cue condition was counterbalanced whereby either the social avatar or the non-social stick condition was shown first with all trials completed prior to seeing the other cue condition. Participants were encouraged to take a break every 28 trials and an enforced break was taken between the two cue type sessions where participants removed the HMD.

A trial proceeded as follows, a fixation cross was presented for 1000 ms (inter trial interval), the cue was then presented looking/ pointing down at the table for 1500 ms. The cue then looked up/ pointed up at the participant (transition 500 ms – with the eyes moving rapidly during the first 30 ms of the head movement – reflecting real gaze behaviour, (e.g. Hayhoe et al., 2012) [7] ), thus in the social gaze condition engaging eye contact, and in the non-social stick condition pointing at the participant. After 1000ms the cue pointed/looked down to the left or right (transition 500 ms: again, for the gaze cue the eyes moved rapidly during the first 30ms), targets were then presented, meaning that the stimulus onset asynchrony (SOA) was 500 ms, calculated from the moment the cue began to shift. All four items were presented for encoding for 500ms in four of six possible locations on either the valid (pointed towards) or invalid (pointed away from) side. After a 1000ms blank maintenance interval the location probe was shown, and participants responded using the touch controller in their dominant hand and received accuracy feedback. Next, the status text probe was shown, this could probe the same item as the location probe or a different item, randomised by the computer, again participants responded using the touch controller and received accuracy feedback. There was no response-window cut off (see Fig. 1). Study and materials can be downloaded here: https://osf.io/s9xmu/files/.

EEG triggers were sent when the avatar or stick appeared, when the objects were presented for encoding, at the start of the maintenance interval, when the location probe was presented, when the participant responded to the location probe, when the status question was presented and when the participant responded to the status probe.

### 2.5 EEG pre-processing

**Fig. 2:**
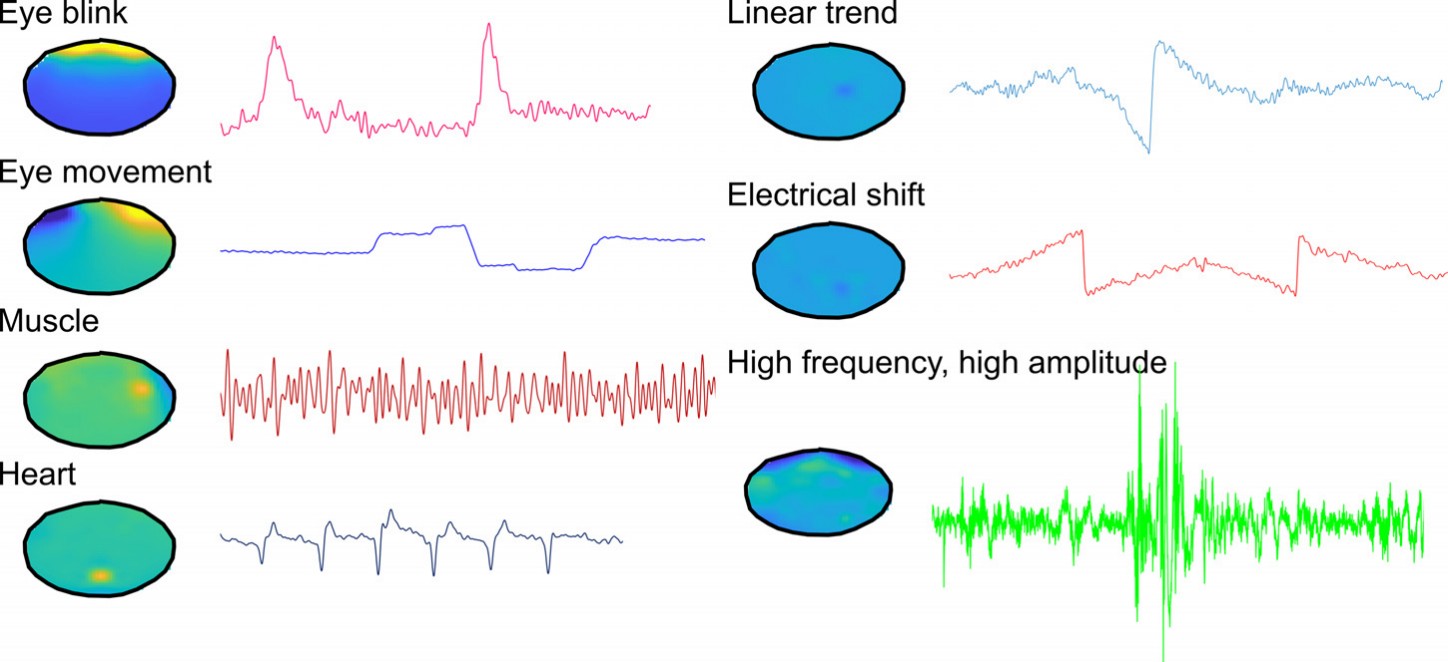
Example artifacts identified through Independent component analysis (fastica).

EEG data were pre-processed using Fieldtrip toolbox version 20191028 [8] in MatlabR2019b®. The pre-processing pipeline went as follows:

1. Data was loaded into Matlab® and channels were selected removing any unused channels, i.e. mastoids and EOG.
2. The data was detrended.
3. The data was bandpass filtered between 0.5 and 36.0Hz.
4. A custom trial function (included in the OpenNeuro repository) was used to epoch the data from 1second pre cue onset to 1 second post probe response (thus including the feedback period), such that cue onset = time 0. This meant that each epoch was at least 7000 ms in length.
5. The practice trials were removed, and accuracy information was added from behavioural data – i.e. whether participants were correct or incorrect for the location and status questions
6. Visual artifact rejection: Trials were visually inspected for artefacts and trials with large artefacts were removed (average 221 total trials per participant included in final analysis). The data was also visually inspected for corrupted electrodes which when identified were interpolated using the average method (5 in total; max 2/ participant).
7. Post visual artifact rejection and interpolation of corrupted electrodes data was re-referenced using the average refence method.
8. Independent component analysis (fastica) was used to identify noise, eye-blink, saccade, heartbeat and muscle components (average 11 components removed per participant, range 2 – 23). This process was manual and examples of the artifacts are presented in Fig. 2.

### 2.6 Behavioural analysis

Behavioural data was analysed using the ANOVA and t test options in standard statistical software, with JASP used to conduct Bayesian analysis, details are included in Gregory et al. [2], behavioural analysis scripts are not provided.

### 2.7 Time frequency analysis

Scripts for the time frequency analysis are provided on OpenNeuro in the code folder; Analysis_MatlabScript_Fieldtrip.txt. The script processes and then analyses the data as follows:

1. The script puts the data into the experimental conditions using ft_selectdata, the key conditions are: 1. All stick (non-social cue) data (StickAll); 2. Congruent stick data (StickCongAll); 3. Incongruent stick data (StickIncongAll); 4. All people (social cue) data (PeopleAll); 5. Congruent people data (PeopleCongAll); 6. Incongruent people data (peopleIncongAll). With the data filtered by response accuracy, as well as without accuracy filtering.
2. A Morlet wavelet transform was performed on each trial using ft_freqanalysis for all channels from 2–30 Hz (for every 1 Hz), with three cycles per time-window in steps of 50 ms with the time window of interest being from the inter trial interval (−0.7) to 1.5 s after the location memory probe was shown (6s).
3. A decibel (db) baseline correction was then applied on the power spectrum field from 500ms to 100ms pre cue onset (−0.5 – −0.1) for each condition using ft_freqbaseline.
4. The grand-average spectra was then computed using ft_freqgrandaverage with keep individual selected to bring the data together for analysis.
5. Analysis was conducted using non-parametric cluster-based permutation tests to correct for multiple comparisons across time points and electrodes. Full parameters are available in the analysis script.

## Ethics Statement

Consent was obtained in accordance with the Declaration of Helsinki and ethical approval was obtained from the Aston University Research Ethics Committee, Protocol number: 1481

## CRediT authorship contribution statement

**Samantha E.A. Gregory:** Conceptualization, Methodology, Software, Validation, Formal analysis, Investigation, Resources, Data curation, Writing – original draft, Supervision, Project administration, Funding acquisition. **Hongfang Wang:** Conceptualization, Methodology, Resources, Software, Writing – review & editing, Project administration. **Klaus Kessler:** Conceptualization, Methodology, Resources, Writing – review & editing, Supervision, Project administration.

## Declaration of Competing Interest

The authors declare that they have no known competing financial interests or personal relationships that could have appeared to influence the work reported in this paper.
